# Ocular Surface Failure in Urban Syndrome

**DOI:** 10.3390/jcm10143048

**Published:** 2021-07-09

**Authors:** Marco Antonini, Daniele Gaudenzi, Sara Spelta, Giancarlo Sborgia, Maria Poddi, Alessandra Micera, Roberto Sgrulletta, Marco Coassin, Antonio Di Zazzo

**Affiliations:** 1 Ophthalmology Operative Complex Unit, University Campus Bio-Medico, 00128 Rome, Italy; m.antonini@unicampus.it (M.A.); d.gaudenzi@unicampus.it (D.G.); s.spelta@unicampus.it (S.S.); maria-poddi@libreo.it (M.P.); r.sgrulletta@unicampus.it (R.S.); m.coassin@unicampus.it (M.C.); 2 Department of Medical Science, Neuroscience and Sense Organs, Eye Clinic, University of Bari, 70121 Bari, Italy; gcsborgia@hotmail.it; 3 Research and Development Laboratory for Biochemical, Molecular and Cellular Applications in Ophthalmological Sciences, IRCCS–Fondazione Bietti, 00198 Rome, Italy; alessandra.micera@fondazionebietti.it

**Keywords:** para-inflammation, ocular surface, glycerophosphoinositol, urban syndrome

## Abstract

Background: Nowadays, the continuous increase in air pollution has significantly changed air quality, leading to the onset of the so-called urban syndrome (US), an allergic-like conjunctivitis triggered by pollutants. These patients are characterized by persistent dysregulation of ocular surface para-inflammation, causing chronic low-grade inflammation and ocular discomfort, with significant consequences for occupational health and job productivity prospects. This study aims to investigate the effects of topical glycerophosphoinositol (GPI) eye drops on the signs and symptoms of US. Methods: A multicenter prospective open interventional study was performed. Patients affected by US, enrolled from occupational medicine clinics, were treated with eye drops containing 0.001% GPI in 0.2% HA vehicle three times a day. Ocular surface disease index (OSDI), tear break-up time (T-BUT), Schirmer test, Oxford score, hyperemia and ocular surface symptoms were recorded at patient enrolment (T0), after 1 week (T1) and after 1 month (T2) of treatment. Results: A total of 113 consecutive patients (226 eyes) were included. OSDI score displayed a significant improvement after one week (T0: 39.9 ± 19, T1: 20.8 ± 17.9, T2: 18.4 ± 15.6, *p* < 0.0001); T-BUT (T0: 5.2 ± 2, T1: 7.7 ± 2.2, T2: 9.7 ± 1.8, *p* < 0.0001) and Schirmer Test (T0: 6.6 ± 2.4, T1: 9.7 ± 2.7, T2: 12.6 ± 2.6, *p* < 0.0001) progressively improved from T0 to T2. Conclusions: trice-daily topic instillation of 0.001% GPI in 0.02% HA vehicle resulted an effective and well tolerated treatment in US patients.

## 1. Introduction

In the ocular surface system, several tissues cooperate in order to maintain the critical homeostasis which allows its ultimate visual function and comfort. This equilibrium is challenged daily by repetitive stimuli and insults, but homeostatic physiologic status is continuously preserved by a strictly regulated innate immune response termed “para-inflammation” [1]. This innate inflammation is self-limiting and directly proportioned to the insult intensity. Aggressive insults, such as infections or tissue injuries, activate para-inflammatory mechanisms, which help the tissue to restore its functionality and homeostasis. A protract or disproportionate para-inflammatory response may induce a shift of homeostatic set point towards inflammatory disease.

Several conditions, such as high-calorie nutrients, low physical activity, exposure to toxic compounds, artificial environment and old age, are associated with dysregulated para-inflammation, leading to low grade inflammation [2,3]. Recently defined clinical entities, like Sick Building Syndrome and Urban Syndrome (US), are common in epidemiological studies of the work environment [4,5]. US may be considered as a transversal, cross-over condition that has some common features of allergy, dry eye, and toxic conjunctivitis, related to poor air conditions and urban environment [6]. The most commonly presented sign of urban conjunctivitis is ocular hyperemia, although mucus discharge is also frequently observed. The main symptoms identified are itching, burning, dryness, photophobia, foreign body sensation and blurry vision.

Persistent dysregulation of para-inflammation results in the inability of the ocular surface to preserve its homeostasis, leading to inflammatory conditions and mild ocular discomfort like in US [7].

Glycerophosphoinositol (GPI) is one of the naturally occurring phosphoinositide metabolites. GPI has been shown to be part of a negative feed-back loop that decreases the IκB kinase α/β, p38, JNK, and Erk1/2 kinase phosphorylation. It also decreases NF-κB translocation and binding to intranuclear inflammatory gene promoters [8,9]. Therefore, it may be considered as an early regulator of inflammatory mechanisms. We believe that 0.001% GPI in 0.02% hyaluronic acid (HA) vehicle eye drops can support ocular surface system in patients affected by US by improving long term ocular surface para-inflammatory response, avoiding pharmacological treatments such as topical steroids and non-steroidal anti-inflammatory drugs (NSAIDs).

## 2. Materials and Methods

In a prospective open interventional study, eligible participants were enrolled by an occupational health clinic to evaluate the safety and efficacy of GPI topical ophthalmic treatment in vehicle with 0.2% HA in patients diagnosed with US.

Inclusion criteria required age ≥18 years, ocular surface disease index (OSDI) score >13 and alterations in clinical tests of ocular surface function (tear break up time (T-BUT) and Schirmer test) in conjunction with signs and symptoms suggestive of US. Particularly, this condition is identified in patients living in urban polluted environment, complaining of ocular discomfort with mild to moderate itching/burning eyes, mild to moderate redness and with nonspecific hyper-reactivity to environmental factors, in which dry eye, allergy and toxic conjunctivitis have been excluded [6]. Exclusion criteria were use of topical or systemic anti-inflammatory drugs within the previous 3 months, previous eye disease or surgery, Sjogren’s syndrome, thyroid disease, use of oral contraceptives, use of any topical ophthalmic treatment (including own artificial tears) within one month and contact lens wearers. The study drug was provided as a sterile 0.2% hyaluronic acid solution with 0.001% GPI; all patients were prescribed to instil 1 drop 3 times a day for one month in both eyes.

The study adhered to the tenets of the Declaration of Helsinki and Ethics committee approval was obtained before the study was started. Written informed consent was obtained from each patient prior to the start of the study.

All the patients have been evaluated in the enrolment visit (T0), after 1 week (T1) and at the end of the study, after 1 month (T2). At every visit were assessed: OSDI questionnaire, T-BUT (represented by the time interval between a complete blink and the appearance of the first black spot in the tear film assessed at slit lamp examination), Schirmer test type I, fluorescein vital staining graded according to Oxford Score, conjunctival hyperaemia graded according to SINK score (0–3). Ocular discomfort symptoms (i.e., foreign body sensation, blurry vision, burning, tearing, itching and photophobia) were also assessed (0–3 score).

A commercial statistical/analytical software program, StatView II (Abacus Concepts, Inc., Barkley, CA, USA), was used for statistical analysis. Statistical analysis was performed by an unpaired *t*-test or ANOVA nonparametric test followed by Tukey-Kramer post hoc analysis for comparison groups. Differences were considered statistically significant at *p* < 0.05. Values are expressed as media ± SD and media SEM (in graphs).

## 3. Results

520 patients affected by US have been screened through the inclusion and exclusion criteria. 113 consecutive patients (226 eyes) were enrolled according to study protocol. The mean age of this study group was 49.1 years, 75 females and 38 males. All patients have been treated with eye drops containing 0.001% GPI in 0.2% HA vehicle three times a day for one month. Mean OSDI score, T-BUT, Schirmer test type 1, Oxford score and Hyperemia score assessed at T0, T1 and T2 are summarized in the following Table 1.

**Figure 1:**
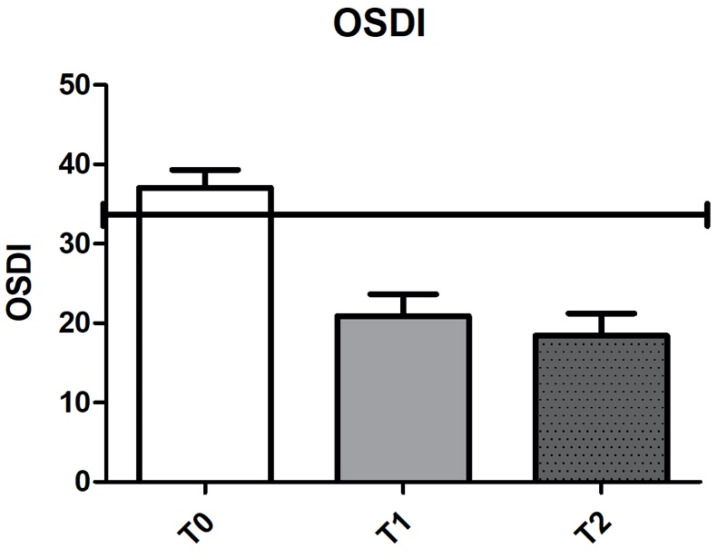
Improvement of symptoms over time recorded via the OSDI questionnaire. The black horizontal line represents the limit value between severe (above) and moderate (below) dry eye. (ANOVA *p* < 0.0001).

**Figure 2:**
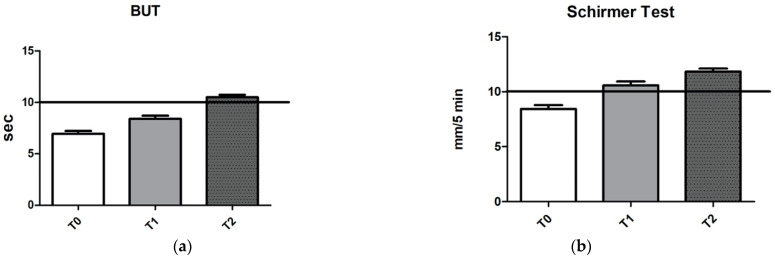
Improvement of tear function over time recorded by both (**a**) TBUT and (**b**) Schirmer’s test. Black horizontal lines represent the boundary between normal (above) and pathological (below) values (ANOVA *p* < 0.0001).

OSDI score, T-BUT and Hyperemia scores are represented in Figure 1 and Figure 2.

**Figure 3:**
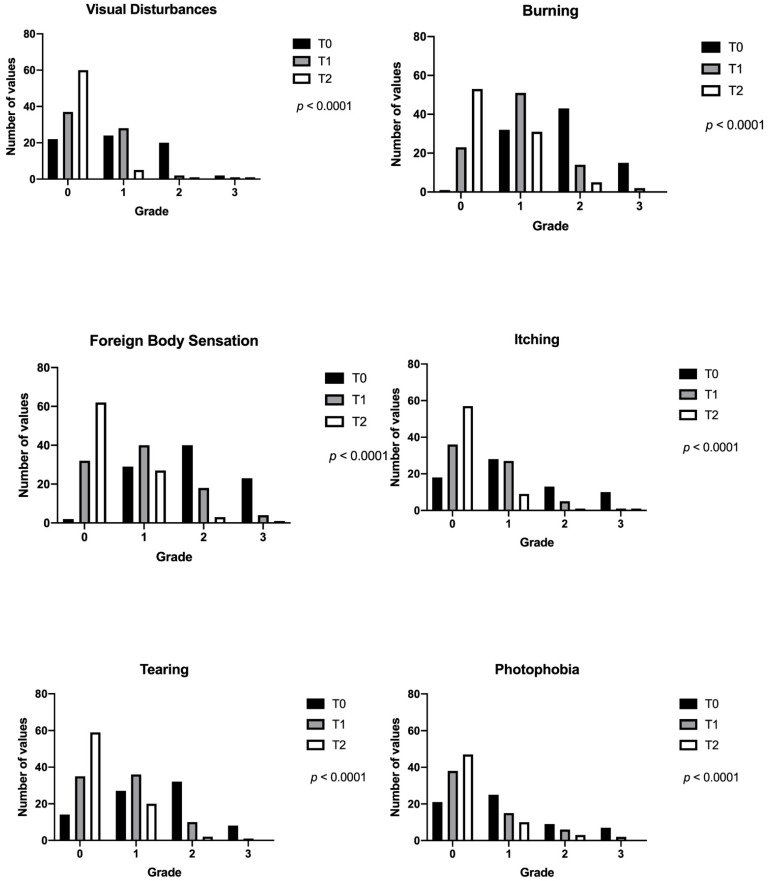
Evolution of the intensity of the several US symptoms at different time frames (ANOVA *p* < 0.0001).

Ocular discomfort symptoms scores are represented in Figure 3.

No significant changes in IOP measurements were observed among patients at different timepoints.

All the patients adhered to therapy and no systemic or local adverse events were reported.

## 4. Discussion

In today’s society, continuously increasing traffic, the ever-growing number of cars and trucks and their resultant fossil fuel emissions and airborne particulates, especially in big cities, along with global warming, have drastically modified air quality. Pollutants have been shown to directly initiate mucosal inflammation through several mechanisms, resulting in non-specific vasomotor conjunctivitis with allergic-like signs and symptoms, without an underlying apparent allergic mechanism and medical history of allergy [6,10].

This condition derives from an ocular surface inability to maintain its own homeostasis in response to non-specific insults. In such patients the ocular response appears excessively severe and unproportionate to environmental insult, leading to a system failure which cause red eye and ocular discomfort symptoms. Since US mostly affects young adults then, it critically impacts the occupational productivity.

Prophylactic, continuous, and constant administration of lubricating eye drops, such as topical administration of 0.001% GPI in 0.02% HA, may be a safe and effective supportive treatment in patients affected by US, training ocular surface system to restore its homeostasis.

In fact, 3 months administration of 0.001% GPI in 0.02% HA 3 times per day improves ocular surface function clinical markers. Indeed, OSDI suddenly reached healthy values after 1 week (Figure 1), while tear film functionality tests, T-BUT and Schirmer test (Figure 2), showed a progressive improvement, reaching a mean value within normal limits at last follow-up visit. These clinical changes express the improved recovery ability of ocular surface system.

In addition, a progressive decrease of symptoms was recorded, emphasizing the subjective benefits felt by the patients treated (Figure 3).

Although the lack of a HA only treated group as further control is a limitation in our study, we still believe that a prophylactic and constant topical lubricating eye drops administration regimen in such population may be effective.

Few reports suggest the use of low-dose corticosteroids in the regulation of para-inflammatory response of the ocular surface. These treatments may be associated with potential side effects, such as increased intraocular pressure, cataracts and infective complications. Therefore, 0.001% GPI in 0.02% HA should be considered a safer alternative to steroids, effective in supporting homeostatic mechanisms of the ocular surface. Therefore, this is a safe first-line treatment for patients suffering by US, which aims to restore homeostatic capabilities of the ocular surface, a matter of public and occupational health concern, especially in the COVID-19 era.

Although the treatment was very successful in reducing ocular symptoms and improving tear functionality outcomes, we found delayed and incomplete resolution of epithelial marks graded on the Oxford scale, perhaps because of the short follow-up.

Clinical data show that the presence of dysregulated para-inflammatory homeostatic mechanisms leads to a strong response to environmental insults, disproportionate to the intensity of the insults themselves. This condition, usually typical of atopic patients, has also been called conjunctival hyperreactivity, i.e., a greater inflammatory susceptibility of some patients to external stimuli, compared to the normal population [11].

Therefore, the para-inflammatory response may be dysregulated in the self-limiting on-off ability, leading to long-term persistent low-grade chronic inflammation, such as in inflammaging [12,13,14], in longstanding diabetes [15], in chronic cicatrizing conjunctivitis [16] or in the intensity of the response itself, such as in US.

Patients suffering from this common disorder may present with severe and acute hyperemia, ocular discomfort and ocular surface function failure in response to mild to moderate insults.

Furthermore, prophylactic administration of 0.001% GPI in 0.02% HA may be suggested in subjects with well-known risk of insufficient ocular surface homeostatic mechanisms, such as screen users, US patients and most of the elderly population [12].

Finally, in the latter elderly population, a steady supply of 0.001% GPI in 0.02% HA may be a valid preoperative eye surface optimization in cataract surgery, decreasing the risk of post-operative ocular discomfort symptoms, the so-called unhappy “20/20” patients [17].

## Figures and Tables

**Table 1:** Average values of the clinical evaluation of dry eye in the study group at different time points.

	Enrolment Visit (T0)	1 Week (T1)	1 Month (T2)	*p*
OSDI	39.9 ± 19	20.8 ± 17.9	18.4 ± 15.6	<0.0001
T-BUT	5.2 ± 2	7.7 ± 2.2	9.7 ± 1.8	<0.0001
Schirmer test type 1	6.6 ± 2.4	9.7 ± 2.7	12.6 ± 2.6	<0.0001
Oxford score	4.8 ± 3.6	4 ± 3.9	2.7 ± 3.5	<0.0001
Hyperemia score	1.8 ± 0.8	1.2 ± 0.8	0.5 ± 0.7	<0.0001
